# Kefir peptides prevent high-fructose corn syrup-induced non-alcoholic fatty liver disease in a murine model by modulation of inflammation and the JAK2 signaling pathway

**DOI:** 10.1038/nutd.2016.49

**Published:** 2016-12-12

**Authors:** H L Chen, T C Tsai, Y C Tsai, J W Liao, C C Yen, C M Chen

**Affiliations:** 1 Department of Bioresources, Da-Yeh University, Changhwa, Taiwan; 2 Department of Life Sciences and Agricultural Biotechnology Center, National Chung Hsing University, Taichung, Taiwan; 3 Graduate Institute of Veterinary Pathology, National Chung Hsing University, Taichung, Taiwan; 4 Department of Internal Medicine, China Medical University Hospital, Taichung, Taiwan; 5 Rong-Hsing Translational Medicine Center, iEGG Center, National Chung Hsing University, Taichung, Taiwan

## Abstract

**Objective:**

In recent years, people have changed their eating habits, and high-fructose-containing bubble tea has become very popular. High-fructose intake has been suggested to be a key factor that induces non-alcoholic fatty liver disease (NAFLD). Kefir, a fermented milk product composed of microbial symbionts, has demonstrated numerous biological activities, including antibacterial, antioxidant and immunostimulating effects. The present study aims to evaluate the effects of kefir peptides on high-fructose-induced hepatic steatosis and the possible molecular mechanism.

**Results:**

An animal model of 30% high-fructose-induced NAFLD in C57BL/6J mice was established. The experiment is divided into the following six groups: (1) normal: H_2_O drinking water; (2) mock: H_2_O+30% fructose; (3) KL: low-dose kefir peptides (50 mg kg^−1^)+30% fructose; (4) KM: medium-dose kefir peptides (100 mg kg^−1^)+30% fructose; (5) KH: high-dose kefir peptides (150 mg kg^−1^)+30% fructose; and (6) CFM: commercial fermented milk (100 mg kg^−1^)+30% fructose. The results show that kefir peptides improve fatty liver syndrome by decreasing body weight, serum alanine aminotransferase, triglycerides, insulin and hepatic triglycerides, cholesterol, and free fatty acids as well as the inflammatory cytokines (TNF-α, IL-6 and IL-1β) that had been elevated in fructose-induced NAFLD mice. In addition, kefir peptides markedly increased phosphorylation of AMPK to downregulate its targeted enzymes, ACC (acetyl-CoA carboxylase) and SREBP-1c (sterol regulatory element-binding protein 1), and inhibited *de novo* lipogenesis. Furthermore, kefir peptides activated JAK2 to stimulate STAT3 phosphorylation, which can translocate to the nucleus, and upregulated several genes, including the CPT1 (carnitine palmitoyltransferase-1) involved in fatty acid oxidation.

**Conclusion:**

Our data have demonstrated that kefir peptides can improve the symptoms of NAFLD, including body weight, energy intake, inflammatory reaction and the formation of fatty liver by activating JAK2 signal transduction through the JAK2/STAT3 and JAK2/AMPK pathways in the high-fructose-induced fatty liver animal model. Therefore, kefir peptides may have the potential for clinical application for the prevention or treatment of clinical metabolic syndrome.

## Introduction

Non-alcoholic fatty liver disease (NAFLD) is the most common factor associated with liver damage. NAFLD results primarily from hepatic steatosis caused by an accumulation of lipids in the liver and may progress toward inflammation with progressive fibrosis.^1^ NAFLD may lead to the development of hepatocellular carcinoma. Currently, three types of NAFLD animal models have been developed, which are classified as genetic, nutritional and a combination of both factors. Nutritional models employ methionine- and choline-deficient, high-fat, high-cholesterol and high-cholate, cafeteria, and high-fructose diets.^2^ Among them, high-fructose and various compositions of high-fat diets have recently been widely studied.^3^ In addition to hepatic steatosis, genetic and nutrition-induced NAFLD animal models result in increased insulin tolerance and NF-kB expression, the main role of which is inflammatory reaction, followed by an increased expression of interleukin (IL)-6 and tumor necrosis factor α (TNF-α) in the liver tissue.^4^ Previous studies have reported that across all gender/age populations, there is an increase in the daily intake of fructose, with previously published estimates of 37 g per day between 1977 and 2004, and 46 g per day between 2007 and 2010.^5, 6^ Obviously, eating habits have shifted toward high-sugar diets during the past decade. Therefore, high-fructose intake is an ideal animal model to mimic the clinical metabolic syndromes.

Kefir grains comprise lactic acid bacteria (*Lactobacillus*, *Lactococcus*, *Streptococcus*, *Enterococcus* and *Leuconostoc*), yeasts (*Kluyveromyces*, *Saccharomyces* and *Torula*), and acetic acid bacteria (*Acetobacter*) and are confined to a matrix of polysaccharides and proteins.^7^ Kefir is traditionally produced by inoculating milk with kefir grain. The literature indicates that kefir is used clinically to treat gastrointestinal disease, hypertension, ischemic heart disease and allergies.^8, 9, 10^ The biological activities of kefir have demonstrated antibacterial, antifungal, antioxidant, anti-diabetic, antitumor and immunostimulating effects.^11^

In the genetic-defect-induced fatty liver disease animal model, we used leptin receptor-deficient ob/ob mice as the NAFLD model to study the preventative effects of kefir peptides in our previous study.^12^ The data demonstrated that kefir improved fatty liver syndrome for body weight, energy expenditure and basal metabolic rate by inhibiting serum glutamate oxaloacetate transaminase and glutamate pyruvate transaminase activities and by decreasing the triglyceride and total cholesterol contents of the liver. In the lipogenesis-related protein expression, kefir markedly decreased the expression of the genes, including sterol regulatory element-binding protein 1 (SREBP-1), fatty acid synthase (FAS) and acetyl-CoA carboxylase (ACC) but not the expression of peroxisome proliferator-activated receptor α (PPARα) or hepatic carnitine palmitoyltransferase-1a in the livers of ob/ob mice.^12^

In this study, we first established a 30% fructose-water-induced NAFLD mouse model to evaluate the effects of kefir peptides. The expression of several genes encoding proteins involved in the lipogenesis pathway, including SREBP-1, FAS and ACC, and in the lipid oxidative pathway, including phospho-Janus kinase 2 (p-JAK2), phospho-signal transducer and the activator of transcription 3 (p-STAT3), phospho-AMP-activated protein kinase (p-AMPK) and phospho-ACC, were measured after 8 weeks of treatment with kefir peptides. The purpose of this study was to understand the efficacy and mechanism of daily administered kefir peptides on anti-inflammation and anti-fatty liver syndrome for further use in modulating or treating NAFLD.

## Materials and methods

### Kefir peptides preparation

Kefir starter grains (Phermpep Co., Taichung, Taiwan) were inoculated (5%, wt/vol) and propagated in sterilized milk according to the previous reports.^12, 13, 14^ After the grains were filtered, the fermented peptide-enriched products were spray-dried as a kefir peptides powder using a spray dryer (Yamato Scientific Co., Tokyo, Japan). The quality controls of kefir peptides powder for the peptides separation and reproducibility are shown in the Supplementary Figure 1. The content of peptides in the kefir peptides powder (Phermpep) calculated as a triglycine equivalent in milligrams per gram within the sample was 21.39 mg g^−1^. The compositions of commercial fermented milk and kefir peptide powder are shown in Supplementary Table 1.

### Animals and study design

Six-week-old male mice (C57BL/6JNarl) were purchased from the National Laboratory Animal Center, Taipei, Taiwan) and kept on a 12-h light–dark cycle at 22±2 ºC. This study was conducted according to institutional guidelines and was approved by the Institutional Animal Care and Utilization Committee of the National Chung Hsing University, Taiwan (IACUC no. 101–97). The animals were given free access to regular rodent chow (a standard laboratory diet) and water *ad libitum*.^15, 16^ After 2 weeks, the C57BL/6 wild-type (WT) mice were randomly divided into the following six treatment groups (*n*=8): (1) normal: H_2_O drinking water; (2) Mock: H_2_O+30% fructose corn syrup; (3) KL: low-dose kefir peptides powder (50 mg kg^−1^)+30% fructose; (4) KM: medium-dose kefir peptides powder (100 mg kg^−1^)+30% fructose; (5) KH: high-dose kefir peptides powder (150 mg kg^−1^)+30% fructose; and (6) CFM: commercial fermented milk (100 mg kg^−1^; LP-33, Uni-President Co., Tainan, Taiwan)+30% fructose. The kefir peptides powder was dissolved in deionized distilled water and orally administered daily for eight weeks. The mice were sacrificed at 16 weeks of age after kefir peptides were administered for the 8-week experiment. All of the animal trials were repeated twice.

### Oral glucose tolerance test

After 8 weeks of kefir peptides administration, an oral glucose tolerance test was performed in all of the groups following an overnight fast (12 h). Briefly, all mice were orally administered glucose (2 g kg^−1^ body weight, 0.1 ml per 10 g body weight; Sigma-Aldrich, St. Louis, MO, USA). The blood glucose levels were measured from the tail vein at 0 (before glucose administration), 30, 60, 90 and 120 min after the glucose loading with a blood glucose meter (Bionime Corp., Taichung, Taiwan).^17^

### Determination of biochemical markers

In sera and liver tissues, alanine aminotransferase (ALT), TG, cholesterol and free fatty acid (FFA) were measured colorimetrically by using an automatic analyzer (Multiskan EX; Thermo Electron Corp., Saint-Herblain Cedex, France). The levels of plasma insulin and hepatic cytokines, including TNF-α, IL-6 and IL-1β, were determined using an ELISA kit (eBioscience, San Diego, CA, USA) according to the manufacturer's protocol.^18, 19^ A homeostasis model assessment for insulin resistance (HOMA-IR) was calculated by the following equation: HOMA-IR index=insulin (μU ml^−1^) × fasting glucose (mmol l^−1^)/22.5.^20^

### Oil Red O staining and hematoxylin and eosin staining

Frozen liver sections (5–7 μm) were stained with Oil Red O and formaldehyde-fixed liver tissues were examined using hematoxylin and eosin (H&E) staining as described previously.^12, 21^ The inflammatory cell infiltration into the liver tissue was evaluated by two pathologists under the high resolution of H&E staining slides.

### Immunohistochemical staining

Formaldehyde-fixed and paraffin-embedded sections were incubated in 3% hydrogen peroxide for 30 min to block endogenous peroxidase activity and then incubated overnight at 4 °C with primary rabbit antibody against 4-hydroxy-trans-2-nonenal (4-HNE; ab46545, Abcam Inc., Cambridge, MA, USA). For antigen retrieval, the sections were immunostained using the VECTASTAIN ABC kit (Vector Laboratories Inc., Burlingame, CA, USA) following the manufacturer's specifications. Diaminobenzidine was used for staining development, and the sections were counterstained with hematoxylin.^12, 22^

### Western blot analysis

The liver tissues were homogenized in 500 μl of a RIPA buffer (Sigma-Aldrich, St. Loius, MO, USA) for protein extraction. The protein (40 μg) was then separated on a 10% SDS-polyacrylamide gel electrophoresis (SDS-PAGE) and electrotransferred onto polyvinylidene difluoride membranes. The membranes were incubated in a blocking solution (5% bovine serum albumin) at room temperature for 2 h and then incubated with a primary antibody (OB-R, SREBP-1, FAS, JAK2, AMPK, ACC, STAT3 or β-actin) overnight at 4 °C. After washing, the membranes were incubated with a goat anti-rabbit immunoglobulin G peroxidase-conjugated secondary antibody. The membranes were developed using an enhanced chemiluminescence western blot detection system (GE Healthcare Biosciences, Pittsburgh, PA, USA) as described previously.^12, 23, 24^

### Statistical analysis

Data were presented as the means±s.e. All data were analyzed using the analysis of variance (ANOVA) test. The analysis was continued with a *post hoc* test using the Duncan method to detect differences in the parameters among the control and treatment groups. Different letters labeled at the top of the columns indicate the significant difference between each group. The threshold for statistical significances was *P*<0.05.

## Results

### Dietary intake, body weight, liver weight and energy intake in fructose-induced NAFLD mice

All of the experimental groups were supplemented with 30% fructose drinking water, including the mock, KL, KM, KH and CFM groups, and showed no significant differences in food and liquid intakes (Supplementary Figure 2). Although the experimental groups exhibited significantly less food and liquid intake compared with the control group that was supplemented with normal drinking water (*P*<0.05), all of the experimental groups showed markedly increased energy intake and body weight compared with the control group. However, treatment with the high-dose of kefir peptides (KH) clearly decreased weight gain compared with the other experimental groups (*P*<0.05). In the liver index (liver weight/body weight ratio), the mock group as a fructose-induced NAFLD mouse model exhibited a significantly higher ratio than the normal control and other experimental groups (Table 1). The results indicated that 30% fructose in the drinking water can induce hepatic steatosis in mice, and kefir peptides can improve the symptoms of NAFLD.

### Effects of kefir peptides on hepatic and serum ALT, TG, cholesterol and FFA

As shown in Table 2, high-fructose supplemented in drinking water clearly increased the contents of hepatic TG, cholesterol and FFA as well as serum ALT, TG in the mock group. However, there were no significant difference in serum cholesterol contents among different groups. Medium- and high-dose treatments of kefir peptides significantly decreased hepatic TG, cholesterol and FFA as well as serum ALT and TG compared with the mock group (*P*<0.05). Moreover, a high-dose of kefir peptides had more efficacy in decreasing hepatic TG content. The medium- and high-dose of kefir peptides also returned the free fatty acids to a normal level.

### Effects of kefir peptides on hepatomegaly and liver injury

**Figure 1:**
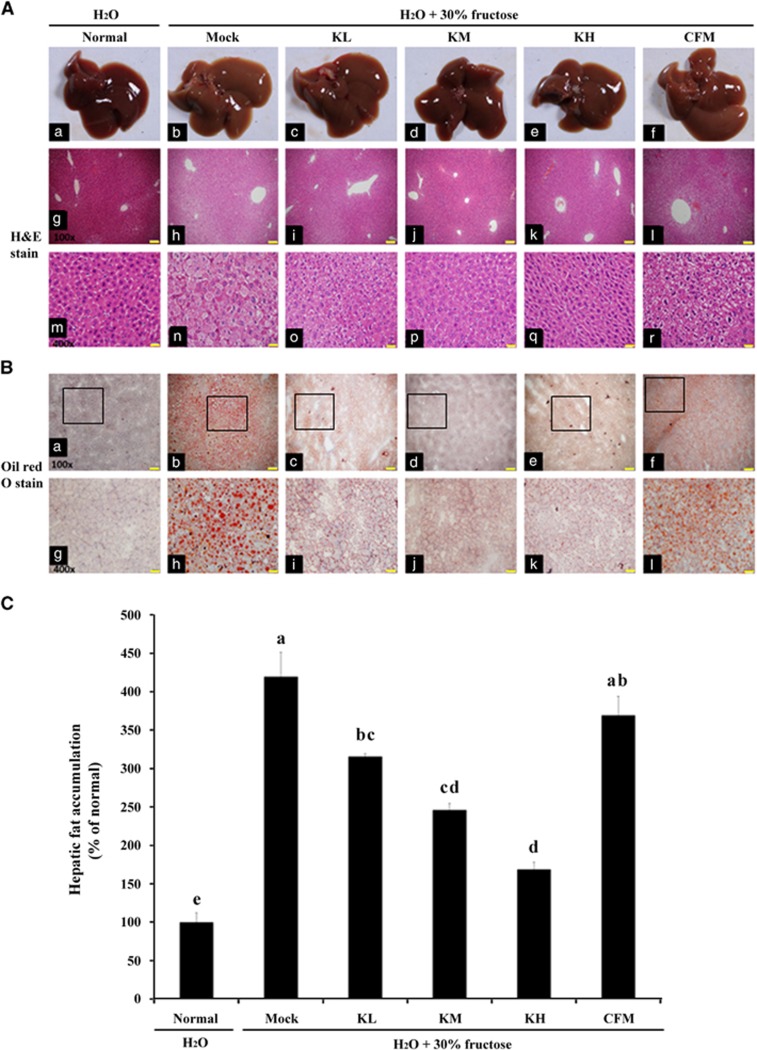
Effects of kefir peptides on the liver pathology and lipid accumulation in 30% fructose-induced NAFLD mice. (**A**) The gross morphology of liver images in different groups of mice was showed in the top panel (a–f). H&E staining of liver sections were represented under 100 × (g–l) and 400 × (m–r) magnification. (**B**) Oil red O staining of liver sections were shown in 100 × (a–f) and 400 × (g–l) representative photomicrographs. Scale bar, 100 μm for 100 × magnification and 20 μm for 400 × magnification. (**C**) Quantitative analysis of hepatic fat accumulation based on lipid droplet counting in Oil red O staining. The values are expressed as the means±s.e. (*n*=8). Different letters labeled in each column indicated a significant difference at *P*<0.05 by one-way ANOVA and Duncan's test.

As shown in Figure 1, the livers of the mock group mice were grossly enlarged and pale in color (Figure 1Aa and b), and their hepatic fat accumulation was also markedly increased (Figure 1Ag and h) compared with the normal control group. Treatment with kefir peptides reduced the formation of fatty liver and lipid accumulation (Figure 1Ai–k and o–q under 100 × and 400 × magnifications, respectively) in these animals in a dose-dependent manner. These histopathological observations were well reflected to the liver weight and liver index of each groups as shown in Table 1. Oil Red O staining of the livers and their quantification data further revealed an accumulation of lipid droplets in the livers of the mock mice group of up to 420% compared with the normal control group, whereas lipid droplets (macrovesicular fat) significantly decreased in the livers of kefir-peptides-treated group mice in a dose-dependent manner, but not in the CFM mice group (Figures 1B and C). These results indicate that treating hepatice steatosis in mice for 8 weeks with kefir peptides decreased the accumulation of liver macrovesicular fat and the symptoms of hepatomegaly.

### Effect of kefir peptides on inflammatory reaction of high-fructose-induced NAFLD mice

**Figure 2:**
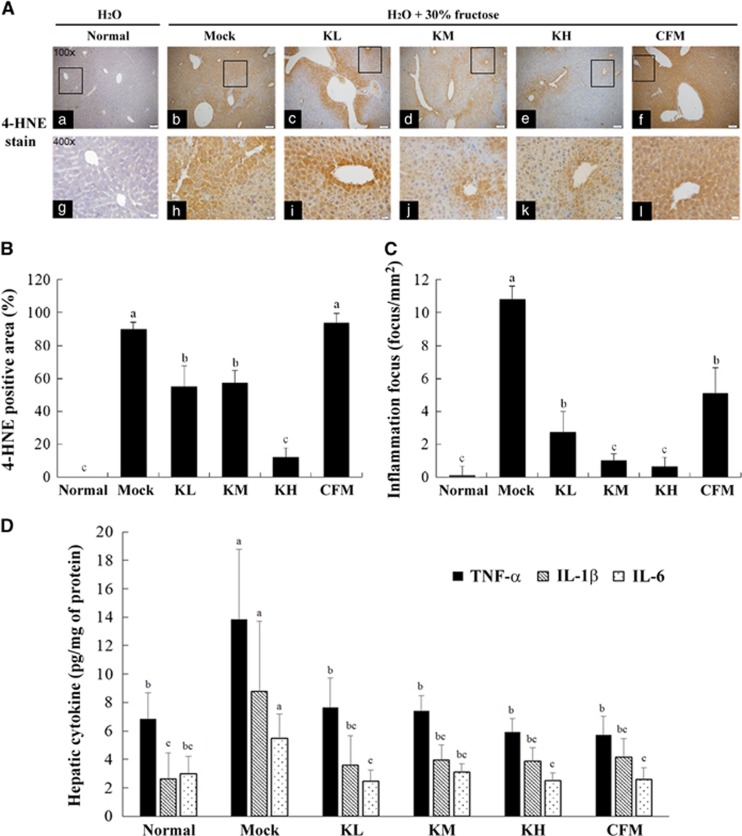
Effects of kefir peptides on the hepatic peroxidation and proinflammatory cytokines in 30% fructose-induced NAFLD mice. (**A**) Immunohistochemical staining of the peroxidation of 4-HNE of liver sections under 100 × (a–f) and 400 × (g–l) magnified photomicrographs. Scale bar, 100 μm for 100 × magnification and 20 μm for 400 × magnification. (**B**) Positively stained cells were quantified in 3 low magnification fields (× 100) of liver sections using ImageJ and the average area of positive cells compared with total cells were calculated. (**C**) Number of inflammation foci (focus per mm^2^) observed in the liver with H&E staining was quantitated by two individual pathologists. (**D**) The hepatic inflammatory cytokines of TNF-α, IL-1β and IL-6 were analyzed by ELISA. The data are expressed as the means±s.e. (*n*=8). Different letters labeled in each column indicated a significant difference at *P*<0.05 by one-way ANOVA and Duncan's test.

Furthermore, 4-HNE is a major product of endogenous lipid peroxidation. High-fructose administration promoted liver cells to express more 4-HNE in the mock mice group (Figure 2Ab and h) and resulted in a liver inflammatory response. Kefir peptides reduced the expression of 4-HNE, particularly in the group of mice treated with a high dose of kefir peptides (Figure 2Ac–e, in 100 × magnification; i–k in 400 × magnification). The positive stained cells were quantified and the average area of positive cells compared with total cells were calculated. The mean of area of Mock, KL, KM, KH and CFM groups were 90.1, 55.1, 37.4, 12.15 and 94.1% respectively (Figure 2B). The inflammatory cell infiltration into the liver tissue was evaluated under the high resolution of H&E staining slides. The inflammation foci were significantly increased in Mock group compared with normal control (*P*<0.05). However, the inflammation foci were significantly reduced by kefir peptides administrations (*P*<0.05) in a dose-dependent manner (Figure 2C). In addition, we measured the inflammation-related cytokines of liver tissues from different experimental mice groups, including TNF-α, IL-1β and IL-6 (Figure 2D). The data showed that the high-fructose intake in the mock group resulted in a high expression of hepatic TNF-α, IL-1β and IL-6 compared with the normal control group (*P*<0.05). Administration of kefir peptides and CFM significantly reduced the expression of those cytokines (*P*<0.05). These results displayed the anti-inflammation potential of kefir peptides as well as CFM products to improve lipid peroxidation and reduce the expression of IL-6, TNF-α and IL-1β cytokine in the liver.

### Effects of kefir peptides on protein expression involved in the JAK2-STAT3 signaling pathway

**Figure 3:**
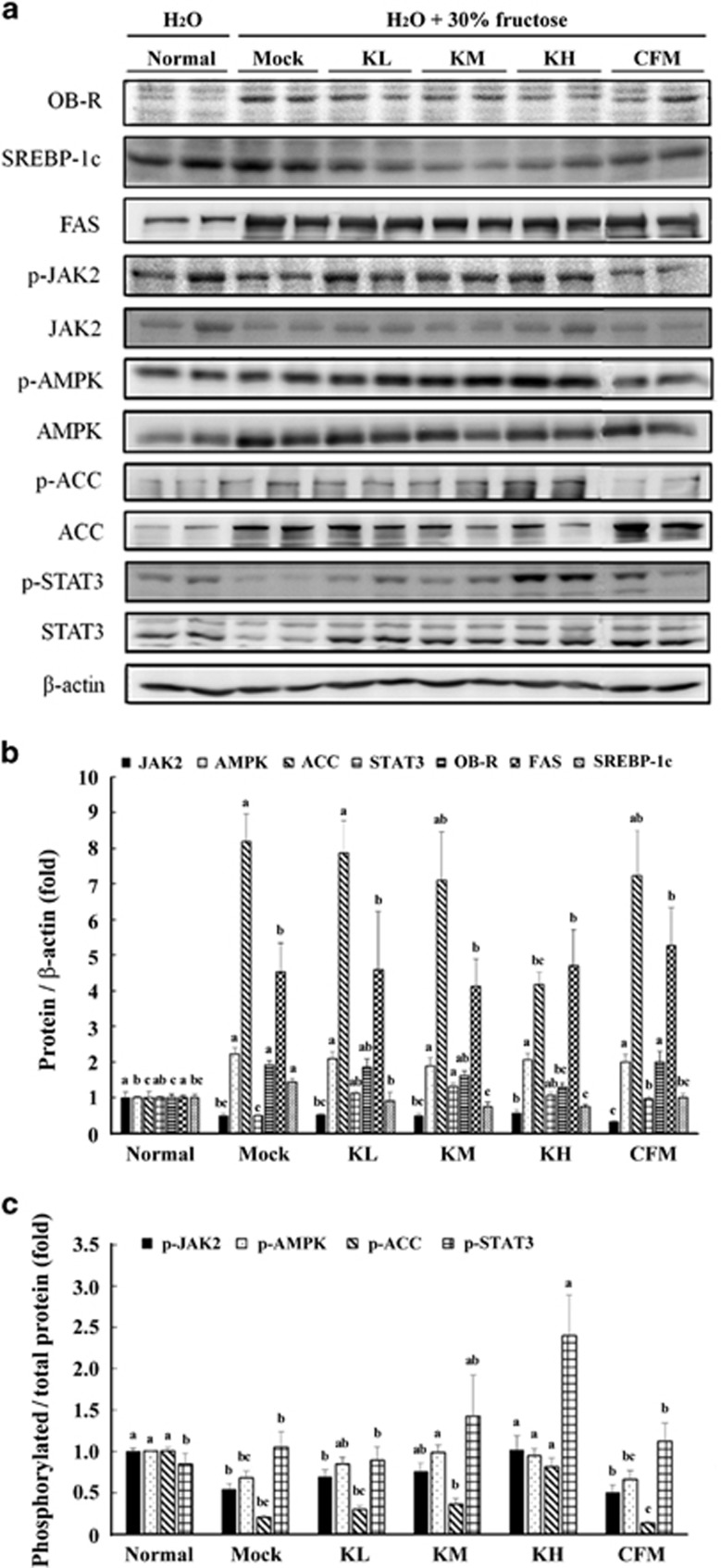
Effects of kefir peptides treatment on the protein expressions involved in the lipid metabolic pathways. (**a**) The expression of lipid metabolic pathway-related proteins including OB-R, SREBP-1, FAS, JAK2, p-JAK2, AMPK, p-AMPK, ACC, p-ACC, STAT3 and p-STAT3 were determined by western blotting. (**b**) Quantitative analysis of relative protein expression levels normalized with the β-actin as an internal control. (**c**) Quantitative analysis of the expression levels of active form phosphorylated proteins, which were normalized with their total protein (*n*=6). Different letters labeled in the same columns indicated a significant difference at *P*<0.05 by one-way ANOVA and Duncan's test.

**Figure 4:**
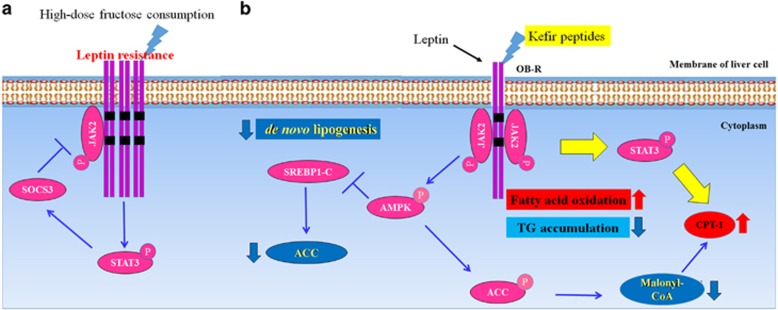
Proposed mechanisms of kefir peptides-mediated modulation of the lipid metabolism pathway in 30% fructose-induced NAFLD mice. (**a**) High-dose fructose consumption resulted in leptin resistance, increasing the expression of LepR (OB-R) and SOCS-3 and blocking of the leptin signal. (**b**) The administration of kefir peptides reduced leptin resistance and activated JAK2 signal transduction through JAK2/STAT3 and JAK2/AMPK pathways to improve the NAFLD syndrome in the fructose-induced fatty liver disease model.

Previous studies have shown that chronic fructose consumption induced leptin resistance before body weight, adiposity, serum leptin, insulin and glucose increases.^25^ Additionally, SOCS-3 expression is induced by leptin receptor (LepR or OB-R) activation, indicating that SOCS-3 is part of a negative feedback loop of the leptin signaling pathway.^26^ LepR transfected CHO cells induced transient expression of endogenous SOCS-3 mRNA, and the forced expression of SOCS-3 resulted in inhibition of leptin-induced tyrosine phosphorylation of JAK2.^27^ Interestingly, LepR transcripts are also expressed in the liver itself,^28^ including the long signaling isoform,^29^ and many studies have revealed that leptin also has direct action on hepatocytes for lipid metabolism.^30^ Figure 3 shows the results of western blotting of the protein expression levels that were involved in lipid metabolism pathways in fructose-induced NAFLD mice. The data shows that SREBP-1c, ACC and FAS were significantly increased in the high-dose fructose-fed mock group (1.4-, 8.1- and 4.5-fold, respectively) compared with normal mice (*P*<0.05). However, fructose-induced NAFLD mice treated with 100 and 150 mg kg^−1^ body weight of kefir peptides (KL and KM, respectively) showed a significant reduction in the expression of SREBP-1 (*P*<0.05), but had no effect on the FAS. Only a high-dose kefir peptides (KH) treatment resulted in an ACC reduction of approximately twofold compared with the mock group (*P*<0.05). High-fructose intake resulted in a significant increase in total AMPK (2.2-fold, *P*<0.05), whereas the p-AMPK/total AMPK ratio was decreased (0.65-fold, *P*<0.05) compared with normal mice. Kefir peptides administration had no effect on total AMPK, but increased the phosphorylation ratio of p-AMPK compared with the mock group (*P*<0.05). In the fatty acid oxidation pathway, high-fructose intake resulted in a significant increase in OB-R compared with normal mice (1.9-fold, *P*<0.05), whereas JAK2, p-JAK2 and p-STAT3 were significantly decreased compared with normal mice (2-, 1.8- and 2-fold, respectively). The data suggest that high-dose fructose consumption resulted in leptin resistance, increasing the expression of OB-R with an expected increase of SOCS-3 and blockade of the leptin signal. Fructose-induced NAFLD mice treated with a high-dose of kefir peptides showed a significant inhibition of the expression of OB-R (1.5-fold) and increased p-JAK2, STAT3 and p-STAT3 levels (1.8-, 2.1- and 2.4-fold, respectively) compared with the mock group. However, the high-dose of kefir peptides administration increased the phosphorylation of p-AMPK and p-ACC and reduced triglyceride accumulation. The proposed mechanism of kefir-peptides-mediated modulation of the lipid metabolism in fructose-induced NAFLD mice through the JAK2 pathway is shown in Figure 4.

### Effects of kefir peptides on glucose tolerance and insulin resistance

**Figure 5:**
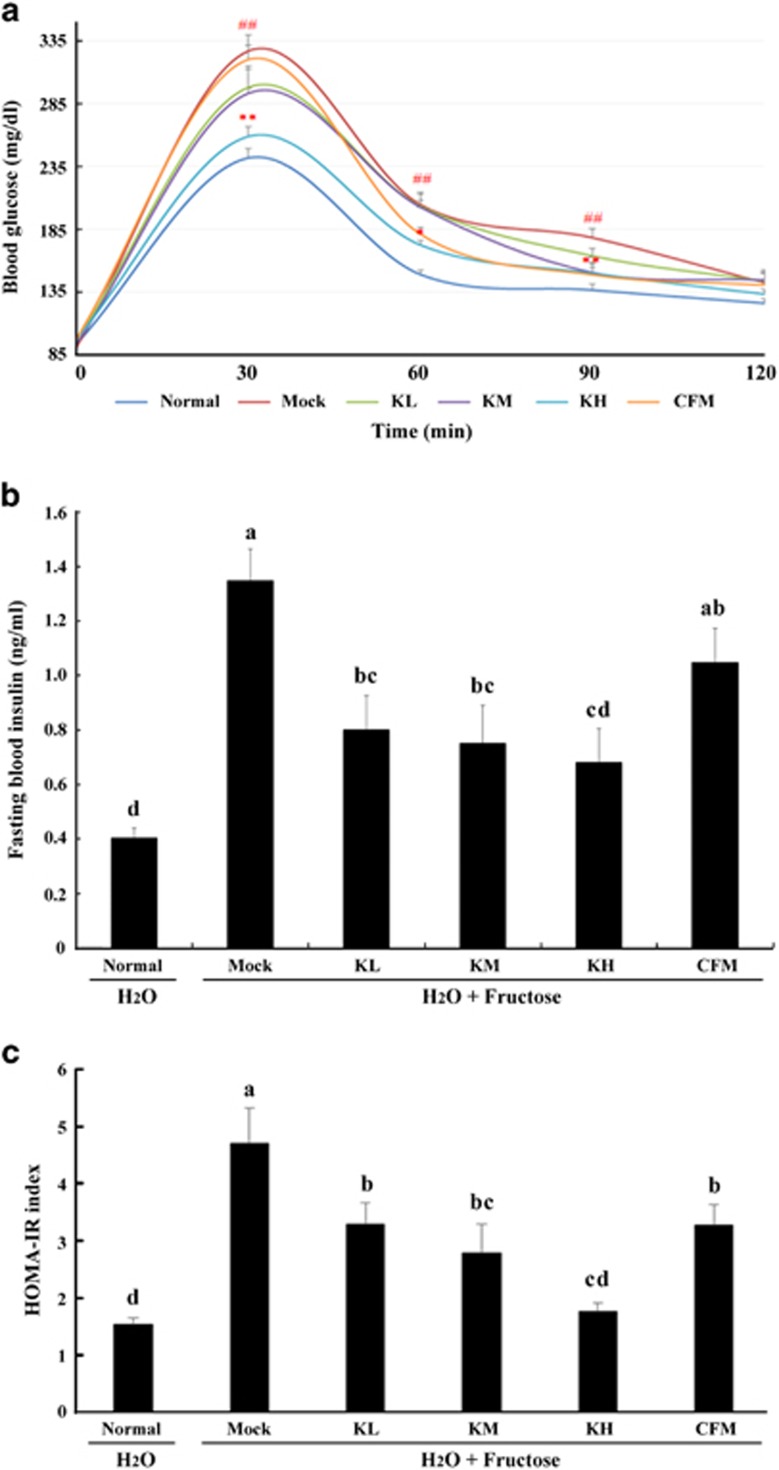
Effect of kefir peptides on the oral glucose tolerance test, insulin resistance, and HOMA-IR index in 30% fructose-induced hepatic steatosis mice after 8 weeks. (**a**) The time-course measurement of blood glucose concentration changes for oral glucose tolerance test. *P<0.05; **P<0.01 vs Mock group; ^##^*P*<0.01 vs normal group. (**b**) The fasting blood insulin concentration and (**c**) the index of homeostasis model assessment for insulin resistance (HOMA-IR). The values are expressed as the means±s.e. (*n*=8). Different letters labeled in each column indicated a significant difference at *P*<0.05 by one-way ANOVA and Duncan's test.

High-fructose corn syrup-induced NAFLD and/or hepatic steatosis is linked with insulin resistance. As shown in Figure 5, measurements of serum glucose by oral glucose tolerance test demonstrated that the mock mice group had higher blood glucose concentrations after fasting and 30, 60 and 90 min post-oral glucose administration compared with the normal mice group (Figure 5a; *P*<0.01). The kefir peptides-treated mice group showed significantly lower blood glucose levels at all times compared with the mock mice group. Fasting insulin levels were significantly reduced in the kefir peptides-treated mice group compared with those of the mock group (Figure 5b; *P*<0.05). The homeostatic model assessment (HOMA) is a method used to detect insulin resistance (HOMA-IR). The data shows that the kefir peptides-treated mice group had significantly reduced HOMA-IR in a dose-dependent manner compared with the mock group (Figure 5c; *P*<0.05). Therefore, kefir peptides treatment could improve glucose tolerance and decrease insulin resistance.

## Discussion

Recent studies have indicated that chronic intake of a 30% fructose solution resulted in a significant 4.2-fold increase in triglycerides and 6.7-fold increase in lipid levels in the liver compared with the normal control group.^31^ It was exhibited as a classic animal model of the non-alcoholic fatty liver disease. In this study, 30% high-fructose drinking consumption resulted in reductions in food intake and liquid intake, but increases in energy intake. This result was consistent with the theory of the dietary compensatory response.^32^ In fact, food intake in fructose-induced hepatic steatosis mice decreased significantly by sixty percent, whereas energy intake increased to twice the energy intake of normal mice. Undernutrition resulted from inadequate food intake, perhaps being one risk of the occurrence of metabolic syndrome in fructose-induced hepatic steatosis mice. The high-fructose-induced NAFLD mice had a significant increase in body weight and the ratio of liver weight to body weight. Although kefir peptides and CFM products cannot improve food intake, they can reduce liver weight, the ratio of liver weight to body weight and hepatic lipid accumulation in fructose-induced hepatic steatosis mice, particularly in the high-dose kefir peptides-treated group. Interestingly, kefir peptides decreased weight gain, but had no effects on food intake. In our previous study, the same kefir peptides product displayed a metabolic rate increase of 39% compared with the ob/ob+ Mock group, the leptin-deficient mice, one of animal model of non-alcoholic fatty liver disease.^12^ In this study, we established another non-alcoholic fatty liver disease animal model induced by feeding 30% high fructose. Therefore, we suggest kefir peptides administration may increase the metabolic rate in these NAFLD models. Those results suggest that high-fructose intake from drinking water resulted in lipodystrophy; however, kefir peptides administration might have improved metabolic rate increasing and hepatic lipid accumulation through inhibited lipogenesis and stimulated lipid oxidation.

Kefir is a fermented milk product comprising a combination of lactic acid and lactose fermentation produced by kefir grains in milk.^33^ The claimed health benefits of kefir include numerous biological activities, such as for the immune system, a reduction of lactose intolerance symptoms, a lowering of cholesterol, and anti-oxidative and anticarcinogenic functions; research on kefir's functional properties has increased during the past decade.^34, 35, 36^ Previous studies have suggested that kefir consumption improves cholesterol metabolism; several hypotheses have been proposed regarding the possible mechanism of action.^37^ However, the biological functions of kefir peptides are largely unknown. In this study, we observed that mice with NAFLD syndrome, which was induced by high-fructose intake, treated with kefir peptides had a significant decrease in hepatic TG, cholesterol, nonesterified free fatty acids, and serum ALT and TG. High-dose kefir peptides had more efficacy in reducing triglycerides in both the liver and the blood. Medium and high doses of kefir peptides restored the nonesterified fatty acids to a normal status.

High-fructose intake was particularly lipogenic in both animals and humans. Daily fructose consumption from food or drinking water has been demonstrated to stimulate *de novo* lipogenesis and results in chronic low-grade inflammation and insulin resistance.^38, 39^ Previous studies have suggested that NAFLD may be associated with an increased formation of reactive oxygen species in the liver and an induction of TNFα.^40^ Recent study showed that lipid peroxidation results in the formation of HNE and upregulates various signaling intermediates that regulate cellular activity and dysfunction via a process called redox signaling. HNE and its glutathione conjugates activate signaling through various protein kinase cascades and regulate NF-B and AP-1, lead to develop a number of inflammatory diseases.^41^ In this study, H&E staining, Oil Red O staining and IHC of 4-HNE of the liver sections revealed an accumulation of lipid droplets and inflammation occurrence in fructose-induced NAFLD mice (Figures 1 and 2). Treating these NAFLD mice with kefir peptides for 8 weeks significantly decreased the accumulation of lipid droplets, the expression of 4-HNE, and some proinflammatory cytokines, including IL-6, TNF-α and IL-1β in the liver tissue. Fructose also activates the hepatic transcription factors carbohydrate-responsive element-binding protein (CREBP), which upregulates the expression of ACC and increases hepatic FAS.^42^ SREBPs regulate the expression of lipogenic enzymes, including ATP-citrate lipase, ACC and FAS. Lipogenesis is controlled by these lipogenic enzymes. The expression of SREBP-1c are increased in fatty livers, and the elevated SREBP-1c increases the expression of lipogenic genes, thereby enhancing fatty acid synthesis and accelerating triglyceride accumulation.^43^ Our previous study showed that *SREBP-1* mRNA and protein expression increased in the livers of the leptin-deficient (ob/ob) mice and that elevated SREBP-1 increased the expression of ACC and FAS, which may accelerate the accumulation of triglycerides and total cholesterol.^12^ In this study, high-fructose intake in the mock group resulted in significant increases in SREBP-1c, ACC and FAS compared with the normal group. However, fructose-induced hepatic steatosis mice treated with 100 and 150 mg kg^−1^ body weight of kefir peptides showed significantly reduced expression of SREBP-1 and had no change in the FAS. Only a high-dose kefir peptides treatment resulted in ACC reduction. Those results suggested that kefir peptides administration could inhibit the lipid accumulation through different signal transduction pathways between fructose-induced hepatic steatosis mice and the leptin knockout mice.

Previous studies have shown that leptin knockout mice (ob/ob) exhibited reduction of the leptin receptor and blocking of the signal transduction of JAK2/STAT3.^44^ However, in this study, high-fructose-induced hepatic steatosis mice showed that the hepatic leptin receptor was significantly increased, while JAK2, p-JAK2 and p-STAT3 were significantly decreased, which means that the JAK2/STAT3 pathway was blocked. Treatment of fructose-induced hepatic steatosis mice with 150 mg kg^−1^ body weight of kefir peptides significantly inhibited the expression of the leptin receptor (OB-R) and increased the expression of p-JAK2, STAT3 and p-STAT3. We suggest that the fructose-induced hepatic steatosis mice exhibited a leptin resistance. However, kefir peptides administration can directly activate the JAK2/ STAT3 pathway to promote fatty acid oxidation. However, AMPD and AMPK are both important in fructose-induced hepatic lipid accumulation in hepatocytes and counter-regulate each other.^45^ Our results demonstrate that high-fructose intake significantly increased the total AMPK expression and decreased the ratio of p-AMPK/AMPK in the liver tissue of NAFLD mice. Kefir peptides administration increased the phosphorylation ratio of p-AMPK and also elevated p-ACC, which will reduce triglyceride accumulation in the liver. Besides, the excess fat accumulation in whole body of fructose-induced NAFLD mice that affect by kefir peptides and results in the decrease of body weight gain may also be an important mechanism. Therefore, we had carry out a new set of experiments to further evaluate the effect of kefir peptides on the lipid metabolism in the adipose tissue of the fructose-induced NAFLD mice.

In conclusion, we demonstrated that chronic fructose intake is associated with hepatic steatosis and inflammation. High-dose fructose consumption also resulted in leptin resistance, increasing the expression of OB-R and blocking the leptin signal (Figure 5a). Fructose-induced hepatic lipid accumulation can be markedly reduced by kefir peptides administration through three possible pathways: (1) kefir peptides significantly reduce the expressions of SREBP-1 and ACC to block lipogenesis; (2) kefir peptides could improve lipid oxidation through reducing the expression of the leptin receptor and increasing the expressions of p-JAK2, STAT3 and p-STAT3; and (3) kefir peptides significantly decrease the inflammatory response and insulin resistance. Therefore, these results suggest that kefir peptides clearly improved the symptoms of NAFLD, by reducing body weight gain, insulin resistance, inflammatory reaction and the formation of fatty liver through the inhibition of the hepatic lipogenesis and the activation of fatty acid oxidation pathway in fructose-induced hepatic steatosis mice.

## Figures and Tables

**Table 1:** Effects of kefir peptides administration on body weight and liver weight in 30% fructose-induced hepatic steatosis mice^1^

*Group*	*Initial body weight (g)*	*Final body weight (g)*	*Weight gain (g)*	*Liver weight (g)*	*Liver weight to body weight ratio (%)*
Normal	19.95±0.49^a^	22.40±0.65^b^	2.45±0.29^b^	0.80±0.03^c^	3.52±0.11^b^
Mock	20.42±0.43^a^	24.31±0.40^a^	4.20±0.16^a^	0.99±0.02^a^	4.06±0.08^a^
KL	20.39±0.19^a^	24.54±0.40^a^	4.18±0.23^a^	0.90±0.01^b^	3.67±0.09^b^
KM	20.47±0.46^a^	24.26±0.50^a^	4.09±0.33^a^	0.87±0.01^b^	3.58±0.07^b^
KH	20.17±0.45^a^	23.24±0.40^a,b^	3.10±0.14^b^	0.84±0.03^b,c^	3.53±0.07^b^
CFM	19.58±0.44^a^	23.86±0.44^a^	4.29±0.45^a^	0.89±0.02^b^	3.72±0.08^b^

Abbreviations: CFM: mice fed with commercial fermented milk (100 mg kg^−1^)+30% fructose; Mock: mice fed with H_2_O+30% fructose; KL, mice fed with low-dose kefir peptides (50 mg kg^−1^)+30% fructose; KM, mice fed with medium-dose kefir peptides (100 mg kg^−1^)+30% fructose; KH, mice fed with high-dose kefir peptides (150 mg kg^−1^)+30% fructose.

1 All of the values are shown as the mean±s.e. (*n*=8).

The different letters presenting in the same column are indicated a significant difference in each group (*P*<0.05).

**Table 2:** Effects of kefir peptides administration on indices of liver in fasting serum and hepatic lipids contents in 30% fructose-induced hepatic steatosis mice^1^

*Group*	*ALT (mg dl^−1^)*	*TG (mg dl^−1^, mg g^−1^)*	*CHOL (mg dl^−1^, mg g^−1^)*	*FFA (μmol g^−1^)*
*Serum*				
Normal	27.9±1.62^c,d^	23.4±3.2^b^	71.1±2.5^a^	
Mock	56.8±5.7^a^	27.9±1.2^a^	73.8±0.8^a^	
KL	40.4±4.3^b,c^	22.4±1.3^b^	71.0±1.5^a^	
KM	37.3±1.9^b,c,d^	22.5±1.2^b^	69.1±2.33^a^	
KH	26.0±2.4^d^	22.3±0.7^b^	71.9±2.04^a^	
CFM	36.9±5.7^b,c,d^	24.8±1.4^a,b^	75.5±2.8^a^	

*Liver*				
Normal		9.5±0.31^e^	10.6±0.35^d^	8.0±0.45^c^
Mock		33.1±3.49^a^	14.9±1.04^a^	15.8±1.88^a^
KL		29.7±1.24^a,b,c^	13.7±0.38^a,b^	12.9±1.59^a,b^
KM		24.1±0.8^c^	12.2±0.34^b,c^	10.4±0.97^b,c^
KH		18.6±1.30^d^	12.1±0.35^c^	9.4±0.80^b,c^
CFM		29.8±1.54^a,b^	13.3±0.34^b,c^	13.0±0.99^a,b^

Abbreviations: ALT, alanine aminotransferase; CHOL, cholesterol; FFA, free fatty acids; TG, triglycerides.

1 All of the values are shown as the mean±s.e. (*n*=8).

The different letters presenting in the same column are indicated a significant difference in each group (*P*<0.05).
