# Supplementary material for: Kefir peptides prevent high-fructose corn syrup-induced non-alcoholic fatty liver disease in a murine model by modulation of inflammation and the JAK2 signaling pathway
Source: Nutr Diabetes. 2016 Dec 12;6(12):e237–. doi: 10.1038/nutd.2016.49 (PMC5223135; doi:10.1038/nutd.2016.49)
Supplement: Supplementary Figure 1 [file nutd201649x1.pdf]

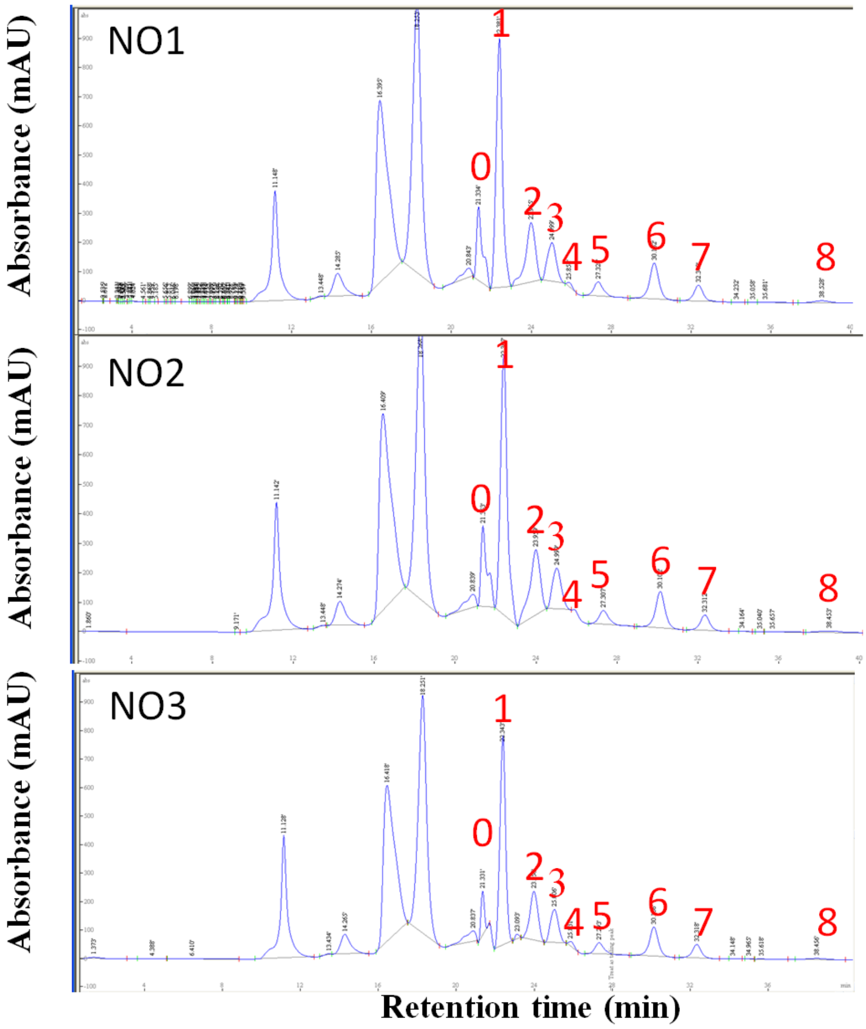


**Supplementary Figure 1. The quality controls of kefir peptides powder for the peptides separation and reproducibility.** They were separated by semipreparative HPLC on a model PU-980 pump (Jasco, Japan) equipped with a UV detector and a 300 x 7.8 mm i.d., 5-µm particles TSK-GEL G2000SWXL column (Sigma-Aldrich, St Louis, MO). The mobile phase was 100 mM KH2PO4, 1 M NaCl and 1 mM EDTA (pH = 6.5) at a flow rate of 0.5 mL/min, and the wavelength was detected at 215 nm.
