# Supplementary material for: Kefir peptides prevent high-fructose corn syrup-induced non-alcoholic fatty liver disease in a murine model by modulation of inflammation and the JAK2 signaling pathway
Source: Nutr Diabetes. 2016 Dec 12;6(12):e237–. doi: 10.1038/nutd.2016.49 (PMC5223135; doi:10.1038/nutd.2016.49)
Supplement: Supplementary Figure 2 [file nutd201649x2.pdf]

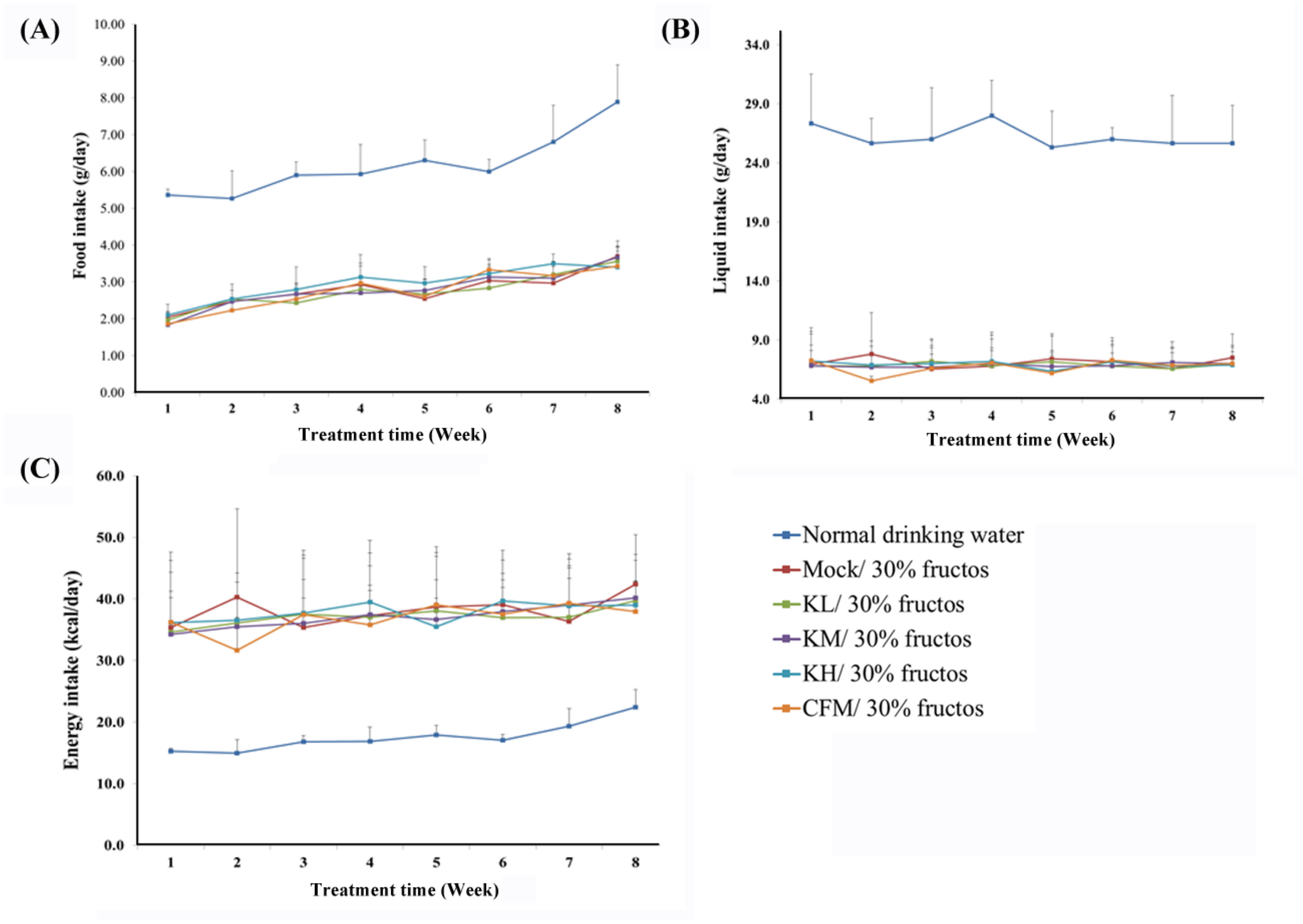

Supplementary Figure 2. Effects of high fructose intake and kefir peptides treatment on daily food intake (A), liquid intake (B) and energy intake (C). Mice were measured, and the food, liquid and energy intakes were calculated weekly during 8 weeks of experimental periods. The data are expressed as the means ± SE (n=8).
