# Supplementary material for: Kefir peptides prevent high-fructose corn syrup-induced non-alcoholic fatty liver disease in a murine model by modulation of inflammation and the JAK2 signaling pathway
Source: Nutr Diabetes. 2016 Dec 12;6(12):e237–. doi: 10.1038/nutd.2016.49 (PMC5223135; doi:10.1038/nutd.2016.49)
Supplement: Supplementary Table 1 [file nutd201649x3.pdf]

Supplementary Table 1. The compositions of commercial fermented milk and kefir peptides powder

Item	Content	
	Commercial fermented milk	Kefir peptides powder
Energy	312 kcal/100g	487 kcal/100g
Protein/peptides	2.79 g/100g	23.1 g/100g
Fat	0.51 g/100g	26.1 g/100g
Carbohydrates	91.9 g/100g	40 g/100g
Sodium	0.11 g/100g	0.28 g/100g
Sugars	49 g/100g	12.7 g/100g
